# The role of age of disclosure of biological origins in the psychological wellbeing of adolescents conceived by reproductive donation: a longitudinal study from age 1 to age 14

**DOI:** 10.1111/jcpp.12667

**Published:** 2016-12-02

**Authors:** Elena Ilioi, Lucy Blake, Vasanti Jadva, Gabriela Roman, Susan Golombok

**Affiliations:** ^1^ Centre for Family ResearchUniversity of CambridgeCambridgeUK

**Keywords:** Egg donation, donor insemination, surrogacy, disclosure, adolescence

## Abstract

**Background:**

The question of whether children should be told of their biological origins is one of the most controversial issues regarding the birth of children through donated eggs, sperm, embryos or surrogacy.

**Methods:**

In the sixth phase of this longitudinal study when the children were aged 14 years, family relationships and adolescent adjustment were examined in 87 families created through reproductive donation and 54 natural conception families. The quality of family relationships was assessed by standardised interview with mothers and by standardised questionnaires and an observational measure with mothers and adolescents. Adolescent adjustment was assessed using standardised questionnaires. Systematic information on whether and when parents had told children about their biological origins was obtained at earlier phases of the study.

**Results:**

There were no overall differences between disclosing families and either nondisclosing or natural conception families. However, within the disclosing families, more positive family relationships and higher levels of adolescent wellbeing were found for adolescents who had been told about their biological origins before age 7.

**Conclusions:**

The earlier children born through reproductive donation are told about their biological origins, the more positive are the outcomes in terms of the quality of family relationships and psychological wellbeing at adolescence.

## Introduction

The question of whether children should be told about their biological origins remains one of the most controversial issues in the practice of reproductive donation, the collective term used to refer to the donation of eggs, sperm or embryos or the hosting of a pregnancy for another woman (surrogacy) (Richards, Pennings, & Appleby, 2012). As a result of these procedures, children may lack a genetic and/or gestational connection to one or both of their parents. Although it is argued by some that children have a right to know their biological origins for both medical and psychological reasons, others believe that this is a private family matter that should be left to parents to decide (Nuffield Council on Bioethics, 2013). Based on research on adoption (Brodzinsky, 2006, 2011) and the family therapy literature (Imber‐Black, 1998; Papp, 1993) as well as reports from donor‐conceived individuals themselves (Turner & Coyle, 2000), there has been a growing shift in opinion towards the view that children born through reproductive donation should be informed of their biological origins.

This change in attitude towards greater openness has resulted in the removal of donor anonymity in some countries so that children born following the introduction of such legislation, and who are aware of their donor conception, may request the identity of their donor on reaching adulthood (Glennon, 2016). In addition, professional guidelines in several countries including the United States and the United Kingdom support the early disclosure of biological origins to children born through reproductive donation (American Society for Reproductive Medicine, 2013; Nuffield Council on Bioethics, 2013).

In spite of these changes to policy and practice, it remains the case that many parents decide against disclosing the use of donor conception to their children (Tallandini, Zanchettin, Gronchi, & Morsan, 2016). Studies on heterosexual parents’ reasons for nondisclosure have shown that they were concerned about jeopardising the positive relationship that had developed between the nongenetic parent and the child and also did not know what, how or when to disclose (Cook, Golombok, Bish, & Murray, 1995; Readings, Blake, Casey, Jadva, & Golombok, 2011). In addition, they were reluctant to disclose their infertility and the involvement of a third party in the birth of their child. Some parents regretted not telling their children but felt that they had left it too late. This is important as there is a growing body of research indicating that the age at which children discover that they were born through donor insemination influences how they feel about the circumstances of their birth, with those who find out later being more likely to experience psychological distress (Jadva, Freeman, Kramer, & Golombok, 2009; Turner & Coyle, 2000). However, these findings are based on retrospective reports of unrepresentative samples, and many of the participants were adults who had not found out about their donor conception until adolescence or beyond.

A major advantage of prospective research on child development is that it enables the impact of a specific event in time to be investigated and allows causal inferences to be made (Robins & Rutter, 1990). The aim of the present study was to investigate the psychological consequences of disclosure to children of their biological origins and of the age at which disclosure took place (Golombok, Lycett, et al., 2004; Golombok, Murray, Jadva, MacCallum, & Lycett, 2004). To this end, systematic information was collected at six time‐points (ages 1, 2, 3, 7, 10 and 14) on whether or not parents had disclosed the nature of their conception to their child and, if so, the child's age at the time of the initial disclosure.

Research on children's developing understanding of biological inheritance has shown that children have an implicit understanding of biological inheritance of physical characteristics by age 4 but it is not until age 7 that they are able to explain this concept and understand the role of genetic mechanisms (Gregg, Solomon, Johnson, Zaitchik, & Carey, 1996; Williams & Smith, 2010). Related to the concept of inheritance, children's development of a biological concept of family emerges at age 7 but an understanding of degrees of biological relatedness is not apparent in the majority of children until age 14 (Richards, 2000; Williams & Smith, 2010). Similar developmental trends have been found in relation to adoption. Although 3‐year‐old children can label themselves as adopted it is not until around 6–7 years that they acquire an understanding of the meaning and implications of adoption, with a more sophisticated understanding developing at adolescence (Brodzinsky, 2011; Brodzinsky & Pinderhughes, 2002). Theoretical approaches to the understanding of adoption emphasise the importance of openness about adoption for positive family relationships and children's psychological wellbeing, especially at adolescence when adolescents need to integrate being adopted into their developing sense of identity (Brodzinsky, 2006; Grotevant, Perry, & McRoy, 2005; Grotevant & Von Korff, 2011).

To the extent that children born through reproductive donation benefit from openness about their origins in the same way as do adopted children (Brodzinsky, 2006; Cahn, 2009; Feast, 2003; Grotevant et al., 2005), less positive mother–child relationships and greater difficulties in child adjustment were hypothesised in the present phase of the study when the children were aged 14 years for families in which children had not been told about their biological origins compared with donor conception families in which children had been told and the comparison group of natural conception families. Furthermore, based on studies showing that the earlier children are informed about their adoption, the better the outcomes in terms of their psychological adjustment and relationships with parents (Brodzinsky & Pinderhughes, 2002; Brodzinsky 2011), it was predicted that adolescents born through reproductive donation who had been told about their biological origins at a young age would show lower levels of emotional and behavioural problems and more positive relationships with their mothers than those told when older. More specifically, it was hypothesised from research on children's developing understanding of inheritance that adolescents who had been told by age 7 would show more positive mother–child relationships and lower levels of adjustment difficulties than those told later, and that those whose parents had started the process of telling by age 3 would show more positive mother–child relationships and lower levels of adjustment problems than those told between age 4 and age 6. Children told at an earlier age have the opportunity to gradually assimilate increasingly complex information about their origins according to their stage of sociocognitive development. In addition, it was hypothesised that the association between age of disclosure and adolescents’ psychological adjustment would be influenced by the quality of family relationships. In particular, based on the finding that openness about adoption is associated with more positive family relationships and more positive adolescent outcomes (Brodzinsky, 2006; Grotevant et al., 2005; Grotevant & Von Korff, 2011), it was predicted that earlier disclosure would be associated with adolescents’ perceptions of more positive family relationships which, in turn, would be associated with greater psychological wellbeing.

## Methods

### Participants

At the previous phase of the study when the children were aged 10 years, parents were asked for permission to contact them again for follow‐up (see Golombok, Lycett, et al., 2004; Golombok, Murray, et al., 2004 for details of the initial recruitment procedures). Those who agreed were approached close to the child's 14th birthday. The present phase involved 87 families with a child born through reproductive donation (32 families with a child born through donor insemination, 27 families with a child born through egg donation and 28 families with a child born through surrogacy), and a comparison group of 54 families with a naturally conceived child, representing 92% of families seen when the children were aged 10 years. Of the surrogacy families, 10 (35.7%) mothers were genetically related to their children as they had used their own eggs to create the pregnancy and 18 (64.3%) lacked a genetic connection as the surrogates’ eggs were used.

The families were categorised into three family types: 31 nondisclosing families; 56 disclosing families and the comparison group of 54 natural conception families. Within the disclosing families, 21 (37%) sets of parents had disclosed by age 3, 25 (45%) had disclosed between ages 4 and 6, and 10 (18%) had disclosed between ages 7 and 14. Data were obtained from all of the mothers in these families. There was no significant difference in age at disclosure between the different types of reproductive donation family, although there was a nonsignificant trend towards earlier disclosure in the surrogacy families. For ethical reasons, it was not possible to obtain data from adolescents who had not been informed of the method of their conception. Thus, all of the naturally conceived adolescents and only those adolescents born through reproductive donation who were aware of the method of their conception were asked to participate. Of the 56 adolescents born through reproductive donation who were invited to participate, 50 (89%) agreed to take part (24 surrogacy adolescents, 16 egg donation adolescents and 10 donor insemination adolescents) and of the 54 natural conception adolescents who were invited to participate, 52 (96%) agreed to take part.

As shown in Table 1, there were no differences between family types in the age or gender of the children. A one‐way analysis of variance (ANOVA) showed that the age of the mothers differed significantly between family types, *F*(2, 138) = 7.76, *p *<* *.001, reflecting the older age of the reproductive donation than natural conception mothers. There was also a significant difference between family types for number of siblings in the family, *χ* ^2^(4) = 22.62, *p *<* *.001, with a greater number of siblings in natural conception than reproductive donation families. There were no differences between family types for the ethnicity or marital status of the mothers. However, the family types differed in the mothers’ educational level, *χ* ^2^(2) = 16.06, *p *<* *.001, reflecting a higher proportion of reproductive donation mothers with a university degree. As the demographic variables that differed significantly between family types were not correlated with the dependent variables, these were not entered into further analyses as covariates.

**Table 1:** Means, *SD*s and *F* values for comparisons between the demographic characteristics of the disclosing, nondisclosing and natural conception families

	Natural conception		Disclosing		Nondisclosing		*F*	*p*
	Mean	*SD*	Mean	*SD*	Mean	*SD*		
Mother's age (years)	48.28	2.74	51.30	5.1	51.19	5.22	7.76	<.001
Child's age (months)	169.17	4.24	167.52	6.21	167.64	5.10	1.61	.204
	*n* (%)		*n* (%)		*n* (%)		*χ* ^2^	*p*
Number of siblings								
0	4 (7)		27 (48)		9 (29)		11.62	<.001
1	41 (76)		23 (41)		18 (58)			
2+	9 (17)		6 (11)		4 (13)			
Child's gender								
Male	25 (46)		26 (46)		19 (61)		2.16	.345
Female	29 (54)		30 (54)		12 (39)			
Mother's education								
No university degree	18 (35)		39 (74)		14 (54)		16.06	<.001
University degree	34 (65)		14 (26)		12 (46)			
Mother's ethnicity								
White	47 (90)		50 (94)		24 (92)		0.58	.752
Not White	3 (19)		3 (6)		2 (8)			
Marital status								
Married/cohabitating	48 (89)		44 (80)		24 (80)		1.89	.394
Separated/divorced	6 (11)		11 (20)		6 (20)			

### Procedure

A psychologist trained in the study techniques visited the families at home. Written informed consent to participate in the investigation was obtained from the mother. Mothers and adolescents also gave written informed consent for the adolescents to participate. Ethical approval for the study was granted by the University of Cambridge Psychology Research Ethics Committee. The mothers were administered a digitally audio‐recorded standardised interview. In addition, the mothers and adolescents completed standardised questionnaires and participated together in a video‐recorded observational task.

### Measures

#### Quality of parenting

##### Interview with mother

The mothers were interviewed using an adaptation of a semistructured interview designed to assess the quality of parenting that has been validated against observational ratings of mother–child relationships in the home (Quinton & Rutter, 1988) and has been used successfully in previous studies of assisted reproduction families (Golombok, Lycett, et al., 2004; Golombok, Murray, et al., 2004). Detailed accounts are obtained of the child's behaviour and the mother's response to it, with particular reference to interactions relating to warmth and control. Examples of questions are: Do you find it easy to be affectionate with (child)? In what ways would you show affection to each other? Does s/he tell you what's bothering her/him or how s/he is feeling? How do you comfort or encourage him/her? Is there any arguing or bad feeling between you? How often does this happen? Do you shout or discuss the issue calmly? How does it end? A flexible style of questioning is used to elicit sufficient information for each variable to be rated by the interviewer using a standardised coding scheme based upon a detailed coding manual. Thus, ratings are carried out by the interviewers using in‐depth information obtained from mothers rather than by the mothers themselves. Some variables are derived from frequency data, such as the frequency of conflict, and the ratings are based on the mother's responses to specific questions. Other variables, such as expressed warmth, are based on information gathered throughout the entire interview and take account of factors such as body language in addition to the content of what is said.

The following variables were coded: expressed warmth from 1 (little) to 5 (high expressed warmth) took account of the mother's tone of voice, facial expressions and gestures in addition to what the mother said about the child; sensitive responding from 1 (low) to 4 (high) represented the mother's ability to recognise and respond appropriately to her child's needs; quality of interaction from 1 (low) to 4 (very high) was based on the extent to which the mother and child enjoyed each other's company; frequency of battles from 0 (never/rarely) to 5 (a few times daily) assessed the frequency of mother–child conflict; level of battles from 0 (none) to 3 (major) assessed the severity of mother–child conflict; and resolution from 0 (full resolution) to 3 (no resolution) assessed the attempt made to resolve the conflict. To establish interrater reliability, 47 interviews randomly selected equally across all family types, representing approximately one third of the families, were coded by a second interviewer. The interviewers were trained in the coding procedure by a researcher who was highly experienced in the coding of this interview (VJ). Training involved the rating of interviews from previous studies with similar samples. In addition, consensus meetings were held for the present study to iron out coding discrepancies. The interclass correlation coefficients were as follows: expressed warmth .70, sensitive responding .56, quality of interaction .79, frequency of battles .99, level of battles .96 and resolution .88. The average intraclass correlation coefficient was .81.

At all phases of the study, a section of the interview focused on issues relating to disclosure including whether or not the parents had decided to tell the child about their biological origins, whether or not they had actually done so, their reasons for or against disclosure, their current feelings in relation to this issue, and the child's response, if told. These variables were coded according to a standardised coding scheme.

#### Global family functioning

##### Index of Family Relationships

Mothers and adolescents completed this 25‐item questionnaire designed to measure problems in family relationships (Hudson, 1989). Sample items are, ‘There is a lot of love in my family’ and ‘There seems to be a lot of friction in my family’. The total score gives an assessment of family relationship difficulties, with higher scores representing greater difficulties. The cut‐off score for clinical problems is 30. Internal consistencies for the original sample ranged from 0.91 to 0.98. The Index of Family Relationships (IFR) has been found to show good discriminant validity and to distinguish between families with and without clinical problems. In the present study, Cronbach alphas for the mothers’ version for the total sample and the natural conception, nondisclosing and disclosing samples, respectively, were .91, .85, .92 and .93, and for the adolescents’ version for the total sample and the natural conception and disclosing samples, respectively, were .93, .93 and .95.

#### Mother–child relationship

##### Parental Acceptance Rejection Questionnaire

The 24‐item version of this questionnaire was administered to both mothers and adolescents to provide total scores of maternal acceptance/rejection, with higher scores representing higher levels of rejection (Rohner, 2001). Examples of items are, ‘I say nice things about my child’ and ‘I hurt my child's feelings’, reworded for the adolescent version. The Parental Acceptance Rejection Questionnaire (PARQ) has been reported to have good internal consistency, with alpha values of .91 and .84, respectively, for the parent and adolescent versions. In the present study, Cronbach alphas for the mothers’ version for the total sample and the natural conception, nondisclosing and disclosing samples, respectively, were .66, .67, .50 and .71, and for the adolescents’ version for the total sample and the natural conception and disclosing samples, respectively, were .82, .84 and .59.

##### Parental Control Scale

This 13‐item measure was completed by mothers and adolescents to provide total scores of behavioural control, with higher scores reflecting higher levels of control (Rohner, 2001). Examples of items are, ‘I let my child go out at any time s/he wants’ and ‘I believe in having a lot of rules and sticking to them’, reworded for the adolescent version. The Parental Control Scale (PCS) has been shown to have good internal consistency with an average Cronbach alpha of .73 from a meta‐analysis of studies using this measure (Rohner & Khaleque, 2003). In the present study, Cronbach alphas for the mothers’ version for the total sample and the natural conception, nondisclosing and disclosing samples, respectively, were .75, .76, .82 and .61, and for the adolescents’ version for the total sample and the natural conception and disclosing samples, respectively, were .84, .85 and .81.

#### Mother–child interaction

##### Observational assessment

Mothers and adolescents participated together in a video‐recorded observational assessment involving a vacation planning task in which they were given 5 min to plan a 2‐week family holiday for which they had unlimited funds (Grotevant & Cooper, 1985, 1986). The session was coded using the Parent–Child Interaction System (Deater‐Deckard & Petrill, 2004) to assess the construct of mutuality, that is, the extent to which the mother and adolescent engaged in positive dyadic interaction characterised by warmth, mutual responsiveness and cooperation. The following variables were rated on a scale ranging from 1 (no instances) to 7 (constant, throughout interaction): Mother's responsiveness *to* child assessed the extent to which the mother responded immediately and contingently to the child's comments, questions or behaviours; Child's responsiveness to mother assessed the extent to which the child responded immediately and contingently to the mother's comments, questions or behaviours; Dyadic reciprocity assessed the degree to which the dyad showed shared positive affect, eye contact and a ‘turn‐taking’ quality of interaction; and Dyadic cooperation assessed the degree of agreement about whether and how to proceed with the task. To establish interrater reliability, 47 interviews randomly selected equally across all family types were coded by two researchers who were unaware of family type. Training involved the rating of video recordings from previous studies with similar samples. The intraclass correlations for child's responsiveness to mother, dyadic reciprocity and dyadic cooperation were .61, .71 and .69 respectively. It was not possible to calculate an intraclass correlation for mother's responsiveness to child as most dyads obtained scores at the top end of the scale.

#### Adolescents' psychological adjustment

##### Strengths and Difficulties Questionnaire

The presence of adolescent psychological problems was assessed with the Strengths and Difficulties Questionnaire (SDQ; Goodman, 2001) administered to mothers and adolescents. The SDQ produces an overall score of adolescent adjustment with higher scores representing higher levels of problems. The cut‐off score for clinical problems is 17 and 20 for mothers and adolescents respectively. Examples of items from the mothers’ questionnaire are, ‘Many fears, easily scared’ and ‘Often fights with other children and bullies them’. Examples of items from the adolescents’ questionnaire are, ‘I am often unhappy, downhearted or tearful’ and ‘I am often accused of lying or cheating’. The SDQ has been shown to have good internal consistency, test–retest and interrater reliability, and concurrent and discriminative validity (Goodman, 2001; Stone, Otten, Engels, Vermulst, & Janssens, 2010). In the present study, Cronbach alphas for the mothers’ version for the total sample and the natural conception, nondisclosing and disclosing samples, respectively, were .58, .66, .55 and .49, and for the adolescents’ version for the total sample and the natural conception and disclosing samples, respectively, were .72, .70 and .75.

##### Engagement, perseverance, optimism, connectedness and happiness measure of adolescent wellbeing

The 20‐item engagement, perseverance, optimism, connectedness and happiness (EPOCH) was administered to adolescents to produce a total score of positive psychological functioning, with higher scores representing more positive functioning (Kern, Benson, Steinberg, & Steinberg, 2016). Sample items are, ‘I am optimistic about my future’ and ‘I have friends I really care about’. Internal consistency has been found to be high, ranging from .85 to .95. EPOCH scores have been shown to be negatively correlated with measures of emotional distress and behaviour problems indicating that the EPOCH is a valid measure of adolescent wellbeing. In the present study, Cronbach alphas for the adolescents’ version for the total sample and the natural conception and disclosing samples, respectively, were .89, .92 and .85.

#### Analysis plan

Confirmatory factor analysis was carried out using the software Mplus v 7.4 (Muthén and Muthén, Los Angeles, CA). Model fit was considered good for CFI and TLI values of ≥.95 and RMSEA values ≤.06 (Bentler, 1992; Hu & Bentler, 1999). Confirmatory factor analysis was conducted with the interview variables relating to parenting quality (CFI = 1.00; TLI = 1.00; RMSEA = .03, 90% CI = .00–.11). Two factors were obtained, each with item loadings of at least 0.43 and all loadings were statistically significant. The first factor (comprising expressed warmth, sensitive responding and quality of interaction) was labelled positive parenting and the second factor (comprising frequency of battles, level of battles and resolution) was labelled negative parenting.

The statistical analyses were carried out in three stages. Firstly, comparisons between the disclosing, nondisclosing and natural conception families were carried out for the mothers’ variables using univariate and multivariate analyses of variance ANOVAs. Secondly, the 56 disclosing families were categorised into three groups according to age at disclosure: 21 families in which parents had initiated disclosure by age 3; 25 families in which parents had initiated disclosure between age 4 and age 6; and 10 families in which parents had initiated disclosure at age 7 or older. Univariate and multivariate ANOVAs were carried out for both the mothers’ and adolescents’ variables to establish whether there were differences according to age at disclosure. Where significant overall differences were found between family types, effect sizes were calculated using the Partial eta squared (*η* ^2^) statistic according to Cohen's criteria (small .01, medium .06 and large .14), and the following Helmert contrasts were conducted (Field, 2013): disclosure before age 7 versus disclosure at age 7 or older to establish whether there were differences according to children's understanding of the concept of inheritance; and disclosure by age 3 versus disclosure between age 4 and age 6 to establish whether there were differences according to children's development of a rudimentary understanding of inheritance. Thirdly, mediation analysis using Mplus v 7.4 was carried out to examine whether the quality of family relationships mediated the effect of age of disclosure on adolescent outcomes.

## Results

### Disclosure versus nondisclosure

As shown in Table 2, MANOVAs were carried out for the variables relating to quality of parenting (positive parenting and negative parenting) and the quality of mother–child relationships (PARQ and the PCS), and univariate ANOVAs were carried out for the variables relating to global family functioning (IFR) and adolescent psychological adjustment (SDQ), with family type (nondisclosing, disclosing and natural conception) as the between‐subjects factor. No significant differences were identified between family types for any of these variables.

**Table 2:** Means, *SD*s and *F* values for comparisons between interview and questionnaire measures of maternal mother–adolescent relationships and adolescent adjustment in disclosed, nondisclosed and natural conception families

	Disclosed		Nondisclosed		Natural conception		*F*	*p*
	Mean	*SD*	Mean	*SD*	Mean	*SD*		
Quality of parenting							1.44	.220
Positive parenting	−0.02	0.78	−0.03	0.69	−0.10	0.88	0.16	
Negative parenting	0.03	0.54	0.16	0.55	−0.09	0.70	1.72	
Global family functioning							0.99	
Mother‐index of family relations	12.11	9.77	12.92	9.84	11.52	7.79	0.22	.806
Measures of mother–child dyad							0.61	.653
Mother: parental Acceptance/Rejection Questionnaire	29.06	4.48	29.63	3.84	29.25	4.25	0.16	
Mother: Parental Control Scale	36.07	4.18	36.67	5.18	35.14	4.82	1.08	
Adolescent psychological adjustment								
Mother: Strengths and Difficulties Questionnaire	5.43	3.39	5.11	3.14	4.47	3.71	1.02	.364

### Age at disclosure

The positive parenting and negative parenting variables from the interview with mothers were entered into a MANOVA with age at disclosure as the between‐subjects factor. Wilks’ *λ* showed a nonsignificant trend, *F*(4, 100) = 2.18, *p *=* *.077, *η* ^2^ = .08, and one‐way ANOVAs showed a significant difference between groups for both positive parenting, *F*(2, 51) = 3.37, *p *=* *.042, *η* ^2^ = .12, and negative parenting, *F*(2, 51) = 3.14, *p *=* *.052, *η* ^2^ = .11. The Helmert contrasts identified more positive parenting and less negative parenting in families where parents had disclosed before the children were 7 years old (positive parenting, *p *=* *.012 and negative parenting, *p *=* *.016) compared to families where parents had disclosed at age 7 or older. No differences were found between families where children had been told by age 3 and families where children had been told between age 4 and age 6 (see Table 3).

**Table 3:** Means, *SD*s, *F* and *p* values for comparisons of interview and questionnaire measures of mother–adolescent relationships and adolescent adjustment and wellbeing in reproductive donation families according to age at disclosure

	By age 3		Between ages 4 and 6		Between ages 7 and 14		*F*	*p*	Contrasts	
									Before 7 versus 7 or older	Before 3 versus between 4 to 6
	Mean	*SD*	Mean	*SD*	Mean	*SD*			*p*	*p*
Quality of parenting							2.18	.077		
Positive parenting	0.13	0.85	0.07	0.76	−0.59	0.48	3.37	.042	.012	.784
Negative parenting	−0.08	0.50	−0.03	0.57	0.40	0.39	3.14	.052	.016	.743
Global family functioning							3.75	.007		
Mother‐index of family relations	8.14	4.79	9.49	7.92	21.20	13.45	7.13	.002	.000	.437
Child‐index of family relations	10.68	8.13	16.03	7.87	21.87	17.53	3.5	.035	.027	.157
Maternal measures of mother–child dyad						2.56	0.043			
Parental Acceptance/Rejection Questionnaire	27.90	2.75	28.50	4.36	32.00	6.50	3.17	.051	.016	.657
Parental Control Scale	36.06	4.08	34.64	4.11	38.20	2.97	2.88	.066	.043	.248
Child measures of mother–child dyad							0.55	.699		
Parental Acceptance/Rejection Questionnaire	27.11	3.41	28.22	3.47	28.78	4.24				
Parental Control Scale	34.61	7.01	34.06	6.80	32.67	3.84				
Adolescent Psychological Difficulties (SDQ)						1.23	0.304			
Mother‐SDQ	5.17	3.22	4.67	2.74	3.67	3.00				
Child‐SDQ	8.39	4.80	10.50	5.86	11.00	5.20				
Adolescent wellbeing (EPOCH)	3.88	0.45	3.76	0.49	3.42	0.61	2.85	.069	.027	.485

With respect to global family functioning, the mothers’ and adolescents’ IFR scores were entered into a MANOVA with age at disclosure as the between‐subjects factor. Wilks’ *λ* was significant, *F*(4, 84) = 3.75, *p *<* *.007, *η* ^2^ = .15. One‐way ANOVAs identified a significant difference between groups for both mothers’ scores, *F*(2, 43) = 7.13, *p *<* *.002, *η* ^2^ = .25, and adolescents’ scores, *F*(2, 43) = 3.53, *p *=* *.035, *η* ^2^ = .14. The Helmert contrasts showed lower mothers’ and adolescents’ scores, reflecting more positive perceptions of family relationships, in families where parents had disclosed before age 7 (mothers, *p *<* *.001 and adolescents, *p *=* *.027) compared to families where parents had disclosed at age 7 or older. There were no differences between families where children had been told by age 3 and families where children had been told between age 4 and age 6.

The mothers’ scores on the PARQ and the PCS were entered into a MANOVA with age at disclosure as the between‐subjects factor. Wilks’ *λ* was significant, *F*(4, 96) = 2.56, *p *=* *.043, *η* ^2^ = .10. One‐way ANOVAs identified a significant difference between groups for the PARQ, *F*(2, 49) = 3.17, *p *=* *.051, *η* ^2^ = .12, and a nonsignificant trend for the PCS, *F*(2, 49) = 2.88, *p *=* *.066, *η* ^2^ = .11. The Helmert contrasts showed higher levels of acceptance (*p *=* *.016) and lower levels of control (*p *=* *.043) in the families where parents had disclosed before age 7 compared to families where parents had disclosed at age 7 or older. No differences were identified between families where children had been told about their biological origins by age 3 and between ages 4 and 6 years. The adolescents’ scores on the PARQ and the PCS were also entered into a MANOVA with age at disclosure as the between‐subjects factor. Wilks’ *λ* was not significant, *F*(4, 82) = 0.55, *p *=* *.699, showing that there were no differences in adolescents’ perceptions of maternal acceptance or control according to age of disclosure.

In addition, the variables relating to the construct of mutuality from the observational assessment of mother–child interaction (mother responsiveness, child responsiveness, dyadic reciprocity and dyadic cooperation) were entered into a MANOVA with age of disclosure as the between‐subjects factor. Wilks’ *λ* was not significant, *F*(8, 72) = 0.30, *p *=* *.963, showing that there was no difference in mother–child interaction according to age at disclosure.

In terms of adolescent adjustment, the mothers’ and adolescents’ SDQ scores were entered into a MANOVA with age at disclosure as the between‐subjects factor. Wilks’ *λ* was not significant, *F*(4, 82) = 1.23, *p *=* *.304, showing that there was no difference in adolescents’ emotional and behavioural problems as rated by mothers or children according to the age at which children had been told about their biological origins. However, a univariate ANOVA with adolescents’ EPOCH scores as the dependent variable and age at disclosure as the between‐subjects factor produced a nonsignificant trend, *F*(2, 42) = 2.85, *p *=* *.069, *η* ^2^ = .12, with the Helmert contrasts showing higher levels of psychological wellbeing among adolescents who had been informed of their biological origins before age 7 (*p *=* *.027) compared to those who had been informed at age 7 or older. There was no difference between children told by age 3 and those told between the ages of 4 and 6 years.

### Mediation analysis

**Figure 1:**
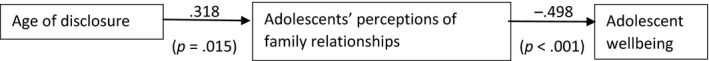
Path analysis for the mediation effect of adolescents’ perceptions of family relationships in the association between age of disclosure and adolescent psychological wellbeing

In order to investigate whether the association between age of disclosure and adolescents’ psychological wellbeing was influenced by their perceptions of the quality of family relationships, a path analysis with indirect effects was carried out. Regression paths were specified from age of disclosure to adolescents’ self‐reported perceptions of family relationships as assessed by the IFR and from adolescents’ perceptions of family relationships to self‐reported wellbeing as assessed by the EPOCH. The direct effect from age of disclosure was not significant and was removed from the model in order to gain 1 degree of freedom and enable the evaluation of model fit. The model, depicted in Figure 1, fitted the data well, with *χ* ^2^(1) = 2.06, *p* = .150 and CFI = .94. The TLI and RMSEA were below acceptable thresholds (Bentler, 1992; Hu & Bentler, 1999) but were excluded from the model fit evaluation as they underperform for models with few degrees of freedom. Thus, earlier disclosure predicted more positive family relationships as perceived by the adolescents, which in turn predicted greater psychological wellbeing. The indirect effect was significant: *β *= −.158, *p* = .039, 95% CI = −.309 −.008.

## Discussion

The findings showed that adolescents who were unaware of their biological origins did not differ from adolescents who had been told about the circumstances of their birth (at any age), or from naturally conceived adolescents, in terms of psychological wellbeing or the quality of family relationships. However, there appears to be variation within families formed through reproductive donation. When the age at which adolescents had learned of their biological origins was examined, more positive family relationships and higher levels of psychological wellbeing were found for adolescents who had been told at a younger age. Specifically, families in which parents had started the process of disclosure before age 7 showed more positive parenting in terms of maternal warmth and sensitivity, and less negative parenting in terms of conflict, as assessed by a standardised interview designed to assess quality of parenting. Moreover, families in which children had been told about their biological origins before age 7 showed higher levels of global family functioning as rated by both mothers and adolescents. Mothers in these families also showed higher levels of acceptance of their adolescent children and lower levels of control although this was not reflected in the adolescents’ perceptions of maternal acceptance or control. No differences were found according to age of disclosure for the observational measure of mother–adolescent interaction.

With respect to the adolescents themselves, there was no difference in psychological problems according to the age of disclosure of their biological origins, as assessed by the SDQ completed by mothers and adolescents. Inspection of the scores showed low levels of emotional and behavioural problems irrespective of their age at the time of disclosure. However, those told earlier showed higher levels of psychological wellbeing as assessed by the EPOCH, with adolescents who learned of their biological origins before age 7 showing higher levels of psychological wellbeing than those who had not been told until age 7 or older. When this finding was explored further, it appeared that earlier disclosure was associated with adolescents’ more positive perceptions of family relationships which, in turn, was associated with higher levels of adolescent wellbeing as assessed by the EPOCH.

Thus, it appears that the earlier children born through reproductive donation are told about their biological origins, the more positive the outcomes in terms of the quality of family relationships and psychological wellbeing at adolescence. The differences based on disclosure relate particularly to mothers’ perceptions of family relationships, suggesting that disclosure may have a greater effect on mothers than on adolescents. The findings are in line with research on adoptive families which has similarly shown that telling children about their adoption at an early age is associated with more positive outcomes for parents and adolescents (Brodzinsky, 2011; Grotevant & Von Korff, 2011; Passmore, Foulstone, & Feeney, 2007; Rueter & Koerner, 2008). The findings are also consistent with research on children's developing understanding of inheritance (Gregg et al., 1996; Richards, 2000; Williams & Smith, 2010). It seems that children born through reproductive donation may be more accepting of information about their biological origins when told by age 7 before they acquire a more complex understanding of the meaning of the absence of a genetic and/or gestational connection to their mother. It cannot be ruled out that the more positive outcomes for early disclosing families were associated with the greater tendency of surrogacy families to disclose at an early age, that is, our findings may have resulted from more positive relationships in the surrogacy families rather than early disclosure. Mothers who have a good relationship with their children may find it easier to be open with them about their origins. Interestingly, no differences were identified between adolescents who had been told of their origins by age 3 and those told between 4 and 6 years old. This suggests that disclosure prior to the increased understanding of inheritance that occurs at around age 4 did not have a long‐term effect on adolescent wellbeing or family relationships.

The finding that the association between age of disclosure and adolescent wellbeing was mediated by adolescents’ perceptions of the quality of family relationships as assessed by the IFR also parallels findings from research on adoptive families which shows that openness about adoption is associated with more positive family relationships and more positive adolescent outcomes (Brodzinsky, 2006; Grotevant et al., 2005; Grotevant & Von Korff, 2011). The IFR assesses perceptions of the family as a whole with questions that are particularly pertinent to adolescents who lack a biological connection to their parents such as, ‘I wish I was not part of this family’ and ‘I feel like a stranger in my family’. Thus, it appears that adolescents born through reproductive donation, like adopted adolescents, benefit from early disclosure of their biological origins by feeling more connected to their family.

From a broader perspective, recent theories of children's sociocognitive and emotional development focus on executive function which refers to cognitive processes such as working memory, inhibitory control and attentional flexibility (Best & Miller, 2010; Garon, Bryson, & Smith, 2008), theory of mind which involves the ability to understand how thoughts and feelings govern human behaviour (Hughes, 2011; Wellman, 2014), and emotional regulation which refers to the processes children use to manage emotions (Holodynski & Friedlmeier, 2006). These theories have shifted away from the earlier Piagetian stage approach to cognitive development towards a more gradual perspective. However, there is evidence that specific transitions in cognitive development do occur. In relation to executive function, for example, it appears that basic skills emerge by age 3, whereas it is not until after age 3 that these skills become coordinated (Garon et al., 2008), and further developmental changes take place at around age 6–7 when children enter school (Best & Miller, 2010). Although the predicted differences between children who first became aware of their biological origins before and after age 3 were not found, the differences identified between children who learned of their origins before and after age 7 may reflect the differences in sociocognitive development that occur at that age. Moreover, once children enter school they may be faced with the challenge of explaining their origins to their peers. This has been shown to present difficulties for children in other new family forms such as children with same‐sex parents (Guasp, Statham, & Jennings, 2010). In relation to adoption, Brodzinsky (2011) highlighted the implications of the changes in cognitive and socioemotional development that take place when children reach school age. In particular, it was argued that children's increased capacity for problem‐solving, logical thought and taking the perspective of others, sensitises them to the reality of adoption‐related loss. Thus, the findings of less positive perceptions of family relationships and lower levels of wellbeing among adolescents who learned of their birth through reproductive donation after age 7 are consistent with changes in children's social understanding at that age.

A limitation of the study was the small sample size. Nevertheless, consistent and meaningful differences relating to the age of disclosure of children's origins were identified from data obtained from both mothers and adolescents, with medium to large effect sizes for all of the significant differences. It should also be noted that the scores for the reproductive donation families compared favourably to normative data for the questionnaire measures. For example, the means for mothers and adolescents for the Index of Family Relations were well below the cut‐off score of 30 for clinical problems, and for the SDQ were well below the clinical cut‐off scores of 17 and 20 for the mothers’ and adolescents’ versions respectively.

A further limitation related to the comparison between the disclosing, nondisclosing and natural conception families. Although no differences were identified between adolescents who had been told about their biological origins and those who had not, data for these comparisons were obtained from mothers only as it was not possible, for ethical reasons, to interview adolescents who were unaware of their biological origins. Moreover, not all of the interview and observational variables showed interrater agreement of 80% or above. The coding of the interview variables that did not reach this threshold involved the use of nonverbal cues such as facial expression and gestures that were not available to the second rater. Thus, the interrater reliabilities of these interview variables may be underestimates. The observational variables showed a restriction in the range of scores rather than poor interrater agreement and have been shown to be reliable in studies of more diverse samples by our own research group (Ensor & Hughes, 2009). Similarly, ceiling effects appeared to account for the moderate alpha values for some of the questionnaires. As the only comparative longitudinal investigation of families formed through reproductive donation, the study provided a unique opportunity to examine the psychological consequences of the age at which children were told about the nature of their conception. An advantage of the study is the use of a multiinformant (mothers, adolescents and teachers) and multimethod (interview, questionnaire and observational assessment) approach. A further strength is that the study has extended findings from the adoption literature to families formed through reproductive donation.

From a theoretical perspective, the findings contribute towards the understanding of the role of openness in families where children lack a biological connection to their parents. Although research on adoptive families has shown that children benefit from communication about their biological origins, adopted children differ from children born through reproductive donation in that they have been relinquished by, or removed from, their birth parents. The situation is somewhat different in families created by reproductive donation where children usually have a genetic and/or gestational connection to one parent and have been raised by their parents from birth. The present study suggests that even under these more favourable circumstances, children benefit from communication about their biological origins from an early age.

From a practical perspective, the findings suggest that parents’ concerns about telling their young children about the circumstances of their birth are unfounded. Indeed, it appears that the earlier that disclosure takes place, the more positive the outcomes for children and their parents. Thus, just as adoptive parents are encouraged be open with their children about their adoption from the start, it seems that parents of children born through reproductive donation should similarly be advised to begin to talk to their children about their origins in their preschool years.

**Key points**

- Whether parents should tell children born through gamete donation or surrogacy about their biological origins is the most contentious issue in the practice of reproductive donation.
- This longitudinal study obtained data from infancy to adolescence on whether and when parents told children born through reproductive donation about their biological origins as well as data on the quality of family relationships and children's psychological adjustment.
- It was found that children told about their origins before age 7 experienced more positive family relationships and higher levels of psychological wellbeing at age 14.
- This suggests that parents should be encouraged to begin to tell their children about their birth through reproductive donation at an early age.
